# The Influence of Maturity Offset, Strength, and Movement Competency on Motor Skill Performance in Adolescent Males

**DOI:** 10.3390/sports7070168

**Published:** 2019-07-09

**Authors:** Andrew W. Pichardo, Jon L. Oliver, Craig B. Harrison, Peter S. Maulder, Rhodri S. Lloyd, Rohan Kandoi

**Affiliations:** 1 Sports Performance Research Institute New Zealand (SPRINZ), AUT Millennium, Auckland University of Technology, 17 Antares Place, Rosedale, Auckland 0632, New Zealand; 2 Youth Physical Development Centre, School of Sport and Health Sciences, Cardiff Metropolitan University, Cyncoed Campus, Cyncoed Road, Cardiff CF23 6XD, UK; 3 Center for Sport Science and Human Performance, Waikato University of Technology, 51 Akoranga Road, Hamilton 3200, New Zealand

**Keywords:** youth, isometric mid-thigh pull, sprint, jump, peak height velocity

## Abstract

This study aimed to examine the extent to which maturity offset, strength, and movement competency influences motor skill performance in adolescent boys. One hundred and eight secondary school boys completed anthropometric and physical testing on two non-consecutive days for the following variables: Maturity offset, isometric mid-thigh pull absolute (IMTP_ABS_) and relative (IMTP_REL_) peak force, resistance training skills quotient, 10-, 20-, and 30-m sprint time, countermovement jump height, horizontal jump distance, anaerobic endurance performance, and seated medicine ball throw (SMBT). The IMTP_REL_ displayed significant small to large correlations with all performance variables (*r* = 0.27–0.61), whereas maturity offset was significantly correlated with IMTP_ABS_ (*r* = 0.69), sprint (*r* = 0.29–0.33), jump (*r* = 0.23–0.34), and SMBT (*r* = 0.32). Absolute and relative strength were the strongest predictors of all performance variables and combined with maturity to explain 21%–76% of the variance. Low and average relative strength boys were nearly eight times (odds ratio: 7.80, confidence interval: 1.48–41.12, *p* < 0.05) and nearly four times (odds ratio: 3.86, confidence interval: 0.95–15.59, *p* < 0.05) more likely to be classified as lower competency compared to high relative strength boys. Relative strength has more influence on motor skill performance than maturity when compared with movement competency.

## 1. Introduction

Motor skill performance during adolescence is influenced by several factors, such as maturation, strength, and movement competency [1,2,3], but the relative importance of each of these factors is currently unknown. Biological maturation, which refers to the process of becoming physically mature [4], is accompanied by large increases in androgenic hormones, lean body mass, stature, and neuromuscular coordination in male youth during the adolescent growth spurt [5]. In European males, this growth spurt occurs between 13–14 years old, with boys growing at a maximum rate of over nine centimeters and over eight kilograms per year [6]. Due to the natural increases in height and muscle mass experienced by males during the growth spurt, strength, and performance in motor skill tasks such as running, jumping, sprinting, and throwing have shown the greatest rates of development during this period [6]. Since the onset and rate of change of these biological changes vary between youth, more physically mature boys are often selected for representative teams [7,8] or viewed as superior to their less mature counterparts of equal chronological age. However, researchers and practitioners can mitigate this bias in a non-invasive way by using somatic measurements to predict peak height velocity (PHV) [9] and adult height [10]. Monitoring biological maturation can provide valuable information to practitioners to better assess and compare youth of a similar chronological age during a period when biological age can vary by as much as five years [4].

Muscular strength can be defined as the ability to produce force [11] and its importance for athletic performance has been noted by several other authors [12,13,14]. It is generally accepted that maturity status and absolute strength are strongly associated, as more mature boys outperform less mature boys during dynamic [15] and isometric strength assessments [16,17]. This is, in part, due to increases in body stature and muscle mass that accompany maturation in males. Relative strength, which accounts for a person’s body mass, may be a better predictor than absolute strength for motor skill tasks such as running and jumping [18,19] since the person must propel their own body mass through space. It is unclear, however, the extent to which relative strength and maturity are independent of one another. Some authors have demonstrated that measures of relative strength are important for running speed, but do not change with advancing age or maturation [20]. However, other authors have suggested that relative strength continues to increase through maturation in boys [21]. Due to the conflicting results from previous studies, further research examining the relationship between maturation and speed is warranted.

The development and reinforcement of movement competency during adolescence is especially important, as some children may experience a temporary loss in coordination during periods of rapid growth [22], a term coined “adolescent awkwardness”. Movement competency refers to an individual’s ability to perform a movement in an optimal manner [23] and is commonly assessed using various screening tools, such as the Resistance Training Skills Battery (RTSB) [24]. Due to the increased movement variability during this phase [25], circa-PHV children are at a heightened risk of injury [26]. Additionally, RTSB scores have been linked to push-up and standing long jump performance [27], as well as cardiorespiratory endurance in youth [28], suggesting movement competency may have both athletic performance and health related implications. However, previous studies have examined the relationship between movement skill and muscular fitness in relatively heterogeneous samples, which potentially inflates the strength of any relationships [24]. For example, Lubans et al. [24] found that RTSB scores explained 39% of the variance in muscular fitness but used both male and females and included a relatively large age range (12–16 years). Other evidence has indicated that maturity and functional movement screen scores influence jump and agility performance in pre- and post-pubertal soccer players [29], excluding circa-pubertal boys. While useful, these findings may not accurately represent the role that movement competency has in motor skill development of circa-PHV males. Therefore, the purpose of this study was to determine the relative contribution of maturity, strength, and movement competency to motor skill performance in running, jumping, and throwing tasks.

## 2. Materials and Methods

### 2.1. Participants

One-hundred and eight circa-PHV males (aged 13–14 years) from a local secondary school in New Zealand volunteered to participate in this study. Descriptive statistics for all participants are shown in Table 1. No participants were injured at the time of testing and all were regularly participating in physical education classes. Parents and participants were informed of the risks and benefits of the study and gave written informed consent and assent, respectively. The project received ethical approval from the University’s Ethics Committee (reference 17/11).

### 2.2. Design and Procedures

A cross-sectional design was used to examine the influence of maturity offset, strength, and movement competency on motor skill performance and was conducted according to the Strengthening the Reporting of Observational Studies in Epidemiology [30]. Testing took place on two non-consecutive days during an hour-long physical education class. Four classes (20–40 students each) were divided evenly into groups of five to seven participants and completed the tests in a randomized order to limit systematic bias. Day one consisted of collecting anthropometric measures, isometric mid-thigh pull (IMTP) peak force, 10-, 20-, and 30-m sprint times, horizontal jump (HJ) distance and countermovement jump (CMJ) height. On the second day, movement competency was assessed using the RTSB and upper body power was measured using the seated medicine ball throw (SMBT). The YoYo Intermittent Recovery Test Level 1 (YYIRTL1) was performed during a separate session the following week. The IMTP and SMBT were conducted by the primary researcher, anthropometric measures were obtained by trained physical education teachers, and several graduate level research assistants conducted the sprint and jump tests. A standardized dynamic warm-up (approximately 10 min) consisting of 10 bodyweight squats, 10 lunges, and 10 push-ups, as well as submaximal jumps and sprints at 50%, 75%, and 90%, was completed prior to each testing session.

#### 2.2.1. Anthropometry

Standing height was measured in centimeters using a stadiometer (Model: WSHRP; Wedderburn, New Zealand). Seated height was measured in centimeters using a meter stick taped to the wall above a 40 cm wooden box. Body mass was measured in kilograms using a digital scale (Model: TI390150K; Tanita, New Zealand). These data were then incorporated into a regression equation to predict maturity offset, which is the length of time (in years) from PHV [9]: Maturity offset = −(9.236 + 0.0002708 × leg length and sitting height interaction)—(0.001663 × age and leg length interaction) + (0.007216 × Age and sitting height interaction) + (0.02292 × weight by height ratio). The Mirwald et al. [9] equation has a standard error of 0.57 years in males and was used because it is a non-invasive method to predict maturation status.

#### 2.2.2. Isometric Mid-thigh Pull

The IMTP was performed using a fixed barbell and two portable force plates (Pasco, CA, USA) sampling at a frequency of 100 Hz and variables were analyzed using custom-built LabVIEW software (version 14.0, National Instruments, Austin, TX, USA). The barbell was fixed in place and the distance between the bar and force plates was adjusted by adding or removing incompressible one-centimeter thick rubber mats until the barbell was positioned just below the hip crease, approximately where the second-pull of a clean starts [11]. Participants used a self-selected mid-thigh clean position with an upright torso (knee angle approximately 125–145°; hip angle approximately 140–150°) [31]. Feet were approximately hip width apart with hands just outside the legs, knees flexed, and torso upright in accordance with previous research [32]. Once the participants were stable in their self-selected positions, a countdown of “3, 2, 1, pull,” was given to initiate the trial. Participants were instructed to pull as hard and as fast as possible for approximately three seconds. Verbal encouragement was given to all participants throughout the trial. Participants performed two maximal trials each with approximately one minute of passive rest between pulls [31]. The trial was discounted and repeated if a countermovement was visible or the participant did not sustain maximal effort for three seconds and the better of the two trials was used for analysis. The maximum force during the pull was reported as absolute peak force (IMTP_ABS_) and was divided by body mass to determine relative peak force (IMTP_REL_).

#### 2.2.3. Resistance Training Skills Battery

Movement competency was assessed using the RTSB, which uses six bodyweight movements: The bodyweight squat, push-up, lunge, suspended row, standing overhead press, and front support with chest touches [24]. Each movement was performed according to the guidelines from Lubans et al. [24], except the bodyweight squat, which included the use of a wooden dowel rod for the squat portion of the assessment. This alteration was used as a more specific tool to assess readiness to back squat. Each movement was filmed from the sagittal and frontal plane with an iPad (3rd and 4th generation, Apple Inc., Cupertino, CA, USA) mounted on a tripod set approximately one meter high and three meters from the center of the capture area. Video assessments were retrospectively played using QuickTime Player (Version 10.4, Apple Inc., Cupertino, CA, USA) and rated according to criteria from Lubans et al. [24]. The push-up and suspended row were rated according to four criteria whereas the other movements were rated according to five criteria. The participant received a “1” for each criterion met or a “0” if they failed to achieve the criteria. The best repetition was scored for each skill. The score from each skill was added together to determine the resistance training skills quotient (RTSQ), which can range from 0–56, with a higher score being better than a lower score.

#### 2.2.4. Sprints

The 10 m sprint time was measured on a wooden gymnasium floor surface using a wired dual-beam infrared system (Swift Performance, Brisbane, Australia). Participants also completed a 30 m sprint outside on an artificial turf surface to determine 20 and 30 m sprint times using a wireless dual-beam infrared system (SpeedLight; Swift Performance, Brisbane, Australia). These tests were conducted separately to mitigate any weather effects on the 10 m sprint. The environmental conditions were the same for all participants when performing the outdoor 30 m sprint (sunny, no heavy wind). Participants used a stationary start 50 cm behind the first timing gate for all sprints. Each participant performed two trials of the 10 and 30 m sprint with at least two minutes rest between trials and the best times were used for analysis. Participants used the same footwear for each testing session.

#### 2.2.5. Horizontal Jump

Participants performed a bilateral horizontal jump with their hands on hips to minimize the effect of arm swing [33,34]. The trial was discounted if the participant’s hands moved from the hips or the feet moved upon landing and therefore another trial was allowed. Jump distance was measured to the nearest centimeter from the furthest back heel using a tape measure secured to the floor. Each participant performed two successful repetitions with at least one-minute rest [35].

#### 2.2.6. Countermovement Jump

The CMJ was performed using a linear position transducer (GymAware; Kinetic Performance Technology, Canberra, Australia) attached to a wooden dowel rod placed across the shoulders in a back-squat position. The subject was instructed to squat down to a self-selected depth and jump as high as possible. Each participant performed two repetitions with at least 30 s rest and the highest jump was used for analysis [36]. The jump height was recorded in centimeters using the GymAware Lite app (Version 2.10, Kinetic Performance Technology, Canberra, Australia) on an iPad (3rd generation; Apple Inc., Cupertino, CA, USA).

#### 2.2.7. Seated Medicine Ball Throw

The SMBT was used to assess upper body power and was measured to the nearest centimeter using a tape measure placed against the wall and taped to the wooden floor of an indoor gymnasium. Participants were instructed to sit with their legs straight and back flat against the wall and hold a 4 kg rubber medicine ball at chest level until instructed to throw. A pause at the chest was used to minimize any momentum or stretch-shortening cycle effects of using a dynamic start. When instructed, the subject threw the ball as far as possible with their back staying in contact with the wall. Each participant performed two throws with at least 30 s rest between throws. The distance was measured from the wall to where the middle of the ball landed, and the best throw was used for analysis.

#### 2.2.8. Yo-yo Intermittent Recovery Test Level 1

The YYIRTL1 was performed in a gymnasium according to the procedures used by Krustrup et al. [37]. The test involved two 20 m runs back and forth at an increasing speed according to an audio recording playing throughout the gym. Each stage was separated by 10 s of active rest consisting of the participants walking five meters, touching a wall, and walking back to the starting line before the next beep. The participant was eliminated when he failed to reach the finish line twice and the total distance covered was recorded and used for analysis. Distance covered in the YYIRTL1 was highly reliable in a group of under-15 males, with CVs below 8% and an ICC of 0.92 [38].

### 2.3. Statistical Analysis

Descriptive data are presented as mean values and standard deviations (SD). A Kolmogrov-Smirnov test confirmed that all variables were normally distributed. Pearson’s product-moment correlation coefficient (*r*) was used to determine relationships between maturity offset, strength, movement competency, and each performance variable. The correlation coefficients were classified according to Hopkins [39]: 0.0–0.1 = trivial, 0.1–0.3 = small, 0.3–0.5 = moderate, 0.5–0.7 = large, 0.7–0.9 = very large, and 0.9–1 = nearly perfect. A stepwise linear regression analysis was used to determine the predictors for the dependent performance variables. The independent variables included maturity offset, IMTP_ABS_, IMTP_REL_, and RTSQ, whereas the dependent variables included the 10, 20, and 30 m sprint time, HJ, CMJ, SMBT, and YYIRL1 for each regression model. To further examine the influence of relative strength on movement competency, an odds ratio (OR) was calculated using binary logistic regression, with participants classified as lower or higher competency based on achieving a RTSQ below or above the group mean. The IMTP_REL_ results were converted to z-scores and participants classified as either low (*z* > −1.0), average (*z* = −1 to 1), or high (*z* > 1) strength. Within-session reliability was calculated using pairwise comparisons on log-transformed data to reduce the effects of any non-uniformity of error [40]. The typical error was expressed as a coefficient of variation (CV) to determine absolute reliability and the intraclass correlation coefficient (ICC) was used to determine relative reliability. All descriptive and reliability data were analyzed using Microsoft Excel 2016 (version 16, Microsoft, Seattle, WA, USA), whereas Pearson correlations, regression analyses, and OR were conducted using SPSS (version 25; SPSS Inc, Chicago, IL, USA) with statistical significance set at *p* ≤ 0.05.

## 3. Results

All tests achieved acceptable (ICC ≥ 0.70 and CV ≤ 15.0%) [25] within-session reliability: IMTP = ICC of 0.93 and CV of 8.3%; 10-m sprint = ICC of 0.93 and CV of 2.1%; 20-m sprint = ICC of 0.97 and CV of 1.5%; 30-m sprint = ICC of 0.97 and CV of 1.5%; HJ = ICC of 0.90 and CV of 4.6%; CMJ = ICC of 0.74 and CV of 13.5%; SMBT = ICC of 0.90 and CV of 6.3%. The RTSB achieved acceptable intra-rater reliability with an ICC of 0.96 and CV of 6.1% after 10 participants were rated and rerated seven days later. Descriptive results for the performance variables of the group are shown in Table 2. The relationships between maturity offset, strength, movement competency, and the motor skill performance variables are shown in Table 3. Maturity offset had a significant, large relationship with IMTP_ABS_ (*r* = 0.69, *p* < 0.01), significant, small to moderate relationships with sprint, jump and throw measures (*r* = 0.23–34, *p* < 0.05), and non-significant, trivial relationships with IMTP_REL_, RTSQ and YYIRTL1 (*r* = 0.00–0.09, *p* > 0.05). The IMTP_ABS_ had significant, large to very large correlations with IMTP_REL_, 30 m sprint, HJ and SMBT (*r* = 0.50–0.82, *p* < 0.01), and moderate correlations with 10- and 20-m sprint, CMJ, and YYIRTL1 (*r* = 0.27–0.49, *p* < 0.01). The IMTP_REL_ had significant, small to large relationships with all performance variables (*r* = 0.27–0.61, *p* < 0.01) and in general had larger correlations with performance variables than IMTP_ABS_. The RTSQ had significant, small to moderate relationships with IMTP_REL_ and running measures only (*r* = 0.21–0.37, *p* < 0.05).

Results of the stepwise linear regression analysis are shown in Table 4. The RTSQ did not significantly contribute to any of the regression models. Maturity offset, IMTP_ABS_, and IMTP_REL_ explained a reasonable amount of the variance for the sprints and SMBT (46%–76%), whereas IMTP_REL_ and maturity offset explained less of the CMJ variance (21%). Strength measures were the only predictors for HJ (IMTP_ABS_ and IMTP_REL_ = 27%) and YYIRTL1 performance (IMTP_REL_ = 26%).

When compared to high strength boys, the low strength boys were nearly eight times more likely to be classified as lower competency (OR = 7.80, 95% confidence intervals [CI] = 1.48–41.21, *p* < 0.05). Although only approaching statistical significance, the average strength boys were nearly four times more likely to be classified as lower competency (OR = 3.86, 0.95–15.59, *p* = 0.058). There was a non-significant increased risk of a low strength boy being classified as lower competency when compared to an average strength boy (OR = 2.02, CI = 0.64–6.35, *p* > 0.05).

## 4. Discussion

This study aimed to examine the influence of maturity offset, strength and movement competency on motor skill performance in a group of 13–14-year-old males. The main finding of the current study suggests that strength is a greater influence than maturity or movement competency on motor skill performance of adolescent boys. Specifically, relative strength generally explains a greater percentage of motor skill performance than absolute strength. Furthermore, strength influences performance more than maturity offset, whereas maturity offset influences performance more than movement competency.

The influence of maturity offset on strength and motor skill performance was apparent in the current study, as evidenced by the significant strong correlations with absolute strength, small to moderate correlations with sprint and jump, and moderate correlations with throw performance. These relationships are similar to previous research on youth males, which have shown significant relationships between maturity and absolute strength [16], speed [17,41,42,43], and jump performance [7,42]. The strength of correlations between maturity offset and a given motor skill may be partially attributed to the increase in body size during PHV. For example, the increase in muscle mass may explain the stronger correlations with IMTP_ABS_ compared to CMJ height. Further, the natural increase in stature and muscle mass during the growth spurt may contribute to increased stride length and therefore faster sprint times, yet may be less beneficial for endurance tasks such as the YYIRTL1. This is reflected by the significant small to moderate correlations between maturity offset and 10–30 m sprints, yet non-significant trivial correlation between maturity offset and the YYIRTL1. Given these findings, maturation may influence speed and endurance performance to different extents. Therefore, practitioners working with youth should understand the extent that maturity offset influences a given fitness quality when identifying talent and designing training programs.

Interestingly, maturity offset was related to IMTP_ABS_, but not IMTP_REL_, suggesting that relative strength measures may be a more useful tool for performance assessment in 13–14-year-old boys, as they do not appear to be influenced by maturation. While maturity offset was not the primary predictor for any of the motor skill tasks, it contributed to predicting sprint, CMJ, and SMBT performance (22%–78%). This suggests that maturation influences performance during adolescence, but not to the extent that strength does. Therefore, measuring variables that account for body mass may be a more effective method to eliminate the maturation bias during common field tests. Furthermore, practitioners should understand the influence maturation can have on motor skill performance when using field tests as selection criteria or for talent identification purposes.

The results from the current study suggest relative strength is the greatest predictor of motor skill performance and displays larger correlations than maturity offset, IMTP_ABS_, or RTSQ with most measures of motor skill performance. These findings support recent research from Meyers et al. [20] which found that greater relative force is associated with step length (*r* = 0.79) and faster sprint speed (*r* = 0.42) in youth males. Furthermore, Thomas et al. [44] showed that relatively stronger athletes outperformed weaker athletes on sprint and jump performance, likely due to the ability to produce more force. Cumulatively, the current study supports findings from existing evidence in confirming the importance of relative strength on motor skill performance. Importantly, the small relationship between RTSQ and IMTP_REL_ was significant, whereas the small relationship with IMTP_ABS_ was non-significant, which suggests the ability to move one’s own body through space is more important than overall force production. The IMTP_ABS_ had the strongest correlation with SMBT performance and explained the most variance, likely due to the same absolute load used for all participants (four kg medicine ball). Despite the relationship between maturity and absolute strength, previous studies indicate measures of relative strength do not improve with increasing chronological age groups in boys [20] or girls [17], or maturity status of girls [45]. Therefore, our findings suggest that developing strength relative to body mass should be a primary goal of long-term athletic development programs, as supported by previous reviews [2,3] and position statements [46]. Physical education teachers can use game-based activities such as tug-of-war, obstacle courses, or partner-based exercises to help develop strength in a fun and engaging manner.

The significant small to moderate correlations between RTSQ and IMTP_REL_, sprint and YYIRTL1 indicate that movement competency is related to measures of relative strength expression, as well as more complex tasks such as sprinting and running. This finding agrees with previous literature that showed associations between measures of movement skill and muscular fitness [24,27,47]. However, there were no significant relationships between RTQS and jump measures in the current study, which may be due to the nature of the assessments. Specifically, jumping performance was assessed bilaterally one repetition at a time, whereas the sprint and YYIRTL1 tests required coordination of contralateral limbs for many rapid, consecutive actions. Thus, moving competently may have a greater influence on performance of complex movements, such as sprinting or sport-specific skills and have less influence on relatively simple tasks, such as a single CMJ or HJ. Furthermore, although correlations between relative strength and competency were only moderate, odds ratio suggests that strength has an important role to play in supporting movement competency. Low and average strength boys were nearly eight and four times more likely to be classified as lower competency, respectively, than high strength boys. This finding highlights the relationship between strength and movement competency and therefore the need for resistance training in adolescence. Nonetheless, motor skill performance is primarily influenced by factors other than movement competency, such as strength and maturity.

A limitation of the current study is that it only included male participants. While males typically experience a neuromuscular spurt from pre- to post puberty, females typically do not and therefore have an increase in knee valgus [48] and landing force, as well as a decrease in jump performance [49]. Given the higher risk of lower-extremity injury in females [48], future research should investigate the relationship between strength and motor skill performance in females. Similarly, future research should aim to investigate the influence of strength on injury risk factors, such as landing kinematics, in adolescent athletes. This information may assist practitioners in developing training programs aimed to reduce the risk of injury in adolescent athletes.

## 5. Conclusions

In conclusion, the current study showed that relative strength is an important factor in differentiating sprint and jump performance in 13–14-year-old boys. Maturity further contributes to performance, but the extent is task dependent and should be accounted for by using relative measures aimed to reduce the influence of body size. The RTSQ was not shown to be a significant predictor of performance in the regression analyses, but had significant relationships with running performance. While relative strength and movement competency do not necessarily naturally improve, previous research has demonstrated the long-term trainability of these physical qualities [21,50,51]. Thus, it is recommended that while youth should be encouraged to train all components of fitness for optimal development [3], a large emphasis should be placed on developing levels of relative strength and movement skill, particularly around PHV. Future research should examine how different training methods improve relative strength, movement competency, and motor skill performance of adolescent males.

## Figures and Tables

**Table 1:** Participant characteristics.

Characteristics	(*n* = 108)
Age (years)	13.9 ± 0.5
Sitting height (cm)	85.9 ± 5.2
Standing height (cm)	166.1 ± 9.4
Body mass (kg)	57.6 ± 13.9
Maturity offset (years from PHV)	0.2 ± 0.9

Values are means and standard deviations.

**Table 2:** Descriptive characteristics of strength and motor performance variables.

Variable	Result
IMTP_ABS_ (N)	924.9 ± 260.2
IMTP_REL_ (N/kg)	16.2 ± 3.3
RTSQ	33.6 ± 7.2
10 m sprint (s)	1.96 ± 0.15
20 m sprint (s)	3.39 ± 0.26 ^a^
30 m sprint (s)	4.82 ± 0.42 ^a^
HJ (m)	1.55 ± 0.21
CMJ (cm)	35.9 ± 7.7
SMBT (m)	3.52 ± 0.67
YYIRTL1 (m)	759 ± 438

Values are means and standard deviations; IMTP_ABS_ = absolute peak force of isometric mid-thigh pull; IMTP_REL_ = relative peak force of isometric mid-thigh pull; RTSQ = resistance training skills quotient; HJ = horizontal jump; YYIRTL1 = Yo-yo Intermittent Recovery Test Level 1; CMJ = countermovement jump; SMBT = seated medicine ball throw; ^a^ = 69 participants.

**Table 3:** Pearson correlations between maturity offset, strength, and RTSQ and motor performance variables.

Variable	Maturity Offset	IMTP_ABS_	IMTP_REL_	RTSQ
IMTP_ABS_	0.69 **			
IMTP_REL_	0.03	0.58 **		
RTSQ	0.00	0.18	0.27 **	
10-m sprint	−0.29 **	−0.45 **	−0.60 **	−0.21 *
20-m sprint	−0.31 *	−0.49 **	−0.61 **	−0.37 **
30-m sprint	−0.33 **	−0.50 **	−0.59 **	−0.37 **
HJ	0.34 **	0.50 **	0.44 **	0.09
CMJ	0.23 *	0.37 **	0.39 **	0.11
YYIRTL1	0.09	0.27 **	0.48 **	0.28 *
SMBT	0.32 **	0.82 **	0.28 **	0.12

IMTP_ABS_ = absolute peak force of isometric mid-thigh pull; IMTP_REL_ = relative peak force of isometric mid-thigh pull (N/kg); RTSQ = resistance training skill quotient; HJ = horizontal jump; CMJ = countermovement jump; YYIRTL1 = Yo-yo Intermittent Recovery Test Level 1; SMBT = seated medicine ball throw; * *p* < 0.05; ** *p* < 0.01.

**Table 4:** Stepwise linear regression analysis of predictors of motor performance.

Dependent Variable	Predictive Variable (s)	R^2^	Adjusted R^2^
10-m sprint	(a) IMTP_REL_	0.40	0.40
	(b) IMTP_REL_, maturity offset	0.47	0.46
	**(c) IMTP_REL_, maturity offset, IMTP_ABS_**	**0.51**	**0.49**
20-m sprint	(a) IMTP_REL_	0.40	0.39
	**(b) IMTP_REL_, maturity offset**	**0.48**	**0.47**
30-m sprint	(a) IMTP_REL_	0.38	0.37
	**(b) IMTP_REL_, maturity offset**	**0.48**	**0.46**
HJ	(a) IMTP_ABS_	0.24	0.23
	**(b) IMTP_ABS_, IMTP_REL_**	**0.28**	**0.27**
CMJ	(a) IMTP_REL_	0.17	0.16
	**(b) IMTP_REL_, maturity offset**	**0.23**	**0.21**
YYIRTL1	**(a) IMTP_REL_**	**0.26**	**0.26**
SMBT	(a) IMTP_ABS_	0.69	0.68
	(b) IMTP_ABS_, IMTP_REL_	0.76	0.75
	**(c) IMTP_ABS_, IMTP_REL_, maturity offset**	**0.77**	**0.76**

IMTP_ABS_ = absolute peak force of isometric mid-thigh pull, IMTP_REL_ = relative peak force of isometric mid-thigh pull; HJ = horizontal jump, CMJ = countermovement jump; YYIRTL1 = Yoyo Intermittent Recovery Test Level 1; SMBT = seated medicine ball throw; all *p* < 0.001. The bold font represents the combination of variables that explains the greatest amount of variance for each performance variable.
